# Supplementary material for: Cryo-EM structure of the RC-LH core complex from an early branching photosynthetic prokaryote
Source: Nat Commun. 2018 Apr 19;9:1568. doi: 10.1038/s41467-018-03881-x (PMC5908803; doi:10.1038/s41467-018-03881-x)
Supplement: Supplementary file 1 — Supplementary Information [file 41467_2018_3881_MOESM1_ESM.pdf]

Supplementary Information

Cryo-EM structure of the RC-LH core complex from an early branching photosynthetic prokaryote

Xin et al.

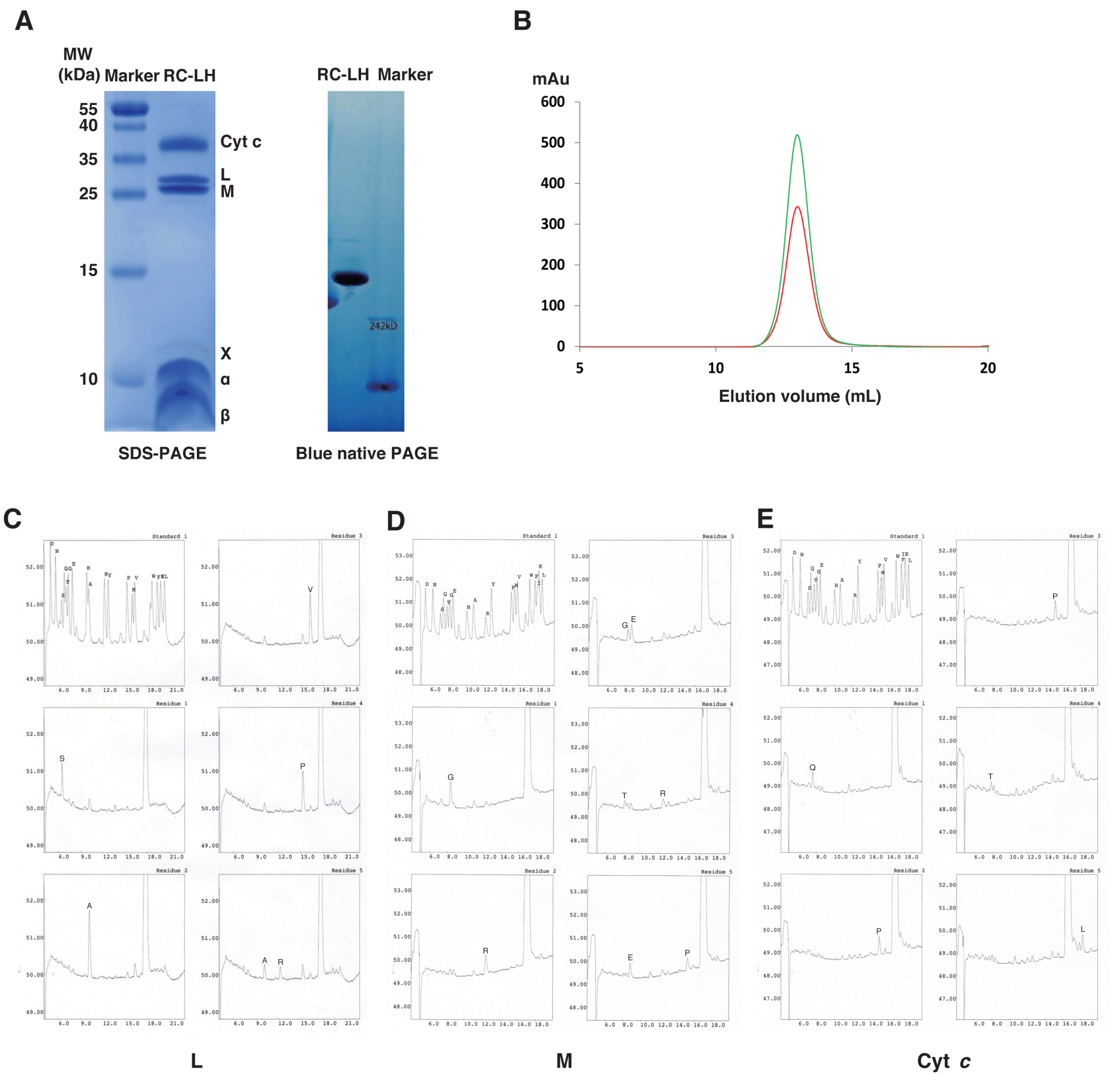

Supplementary Figure 1 | Purification and verification of RC-LH complex. A, SDS-PAGE (left) and blue native PAGE (right) of the RC-LH complex from *R. castenholzii*. For the uncropped images of these gels, please refer to Supplementary Figure 8. **B,** A representative gel-filtration chromatography of RC-LH complex. The chromatography diagram of absorption at 374 nm (green) and 280 nm (red) of the RC-LH complex is shown. **C, D and E,** N-terminal sequencing results of the L (C), M (D) and Cyt *c* subunit (E) of RC-LH complex.

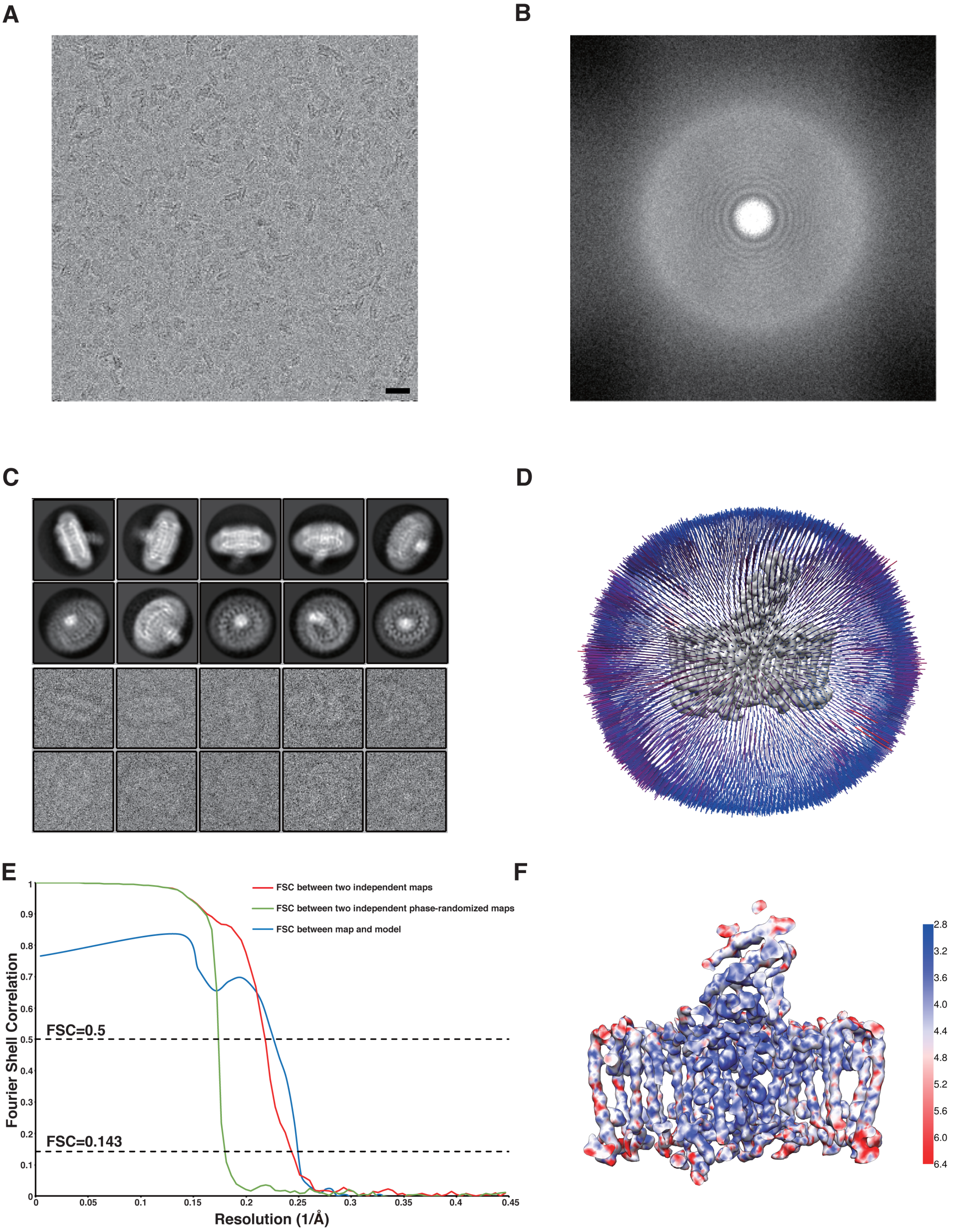
A
B
C
D
E
F
FSC between two independent maps
FSC between two independent phase-randomized maps
FSC between map and model
FSC=0.5
FSC=0.143
Fourier Shell Correlation
Resolution (1/Å)
2.8
3.2
3.6
4.0
4.4
4.8
5.2
5.6
6.0
6.4

Supplementary Figure 2 | Cryo-EM analysis of RC-LH complex. A, A representative raw cryo-EM micrograph of the complex recorded from FEI Falcon IIIEC camera in counting mode (model C). Scale bar, 20 nm. **B,** The power spectrum of (**A**). **C,** Representative reference-free 2D class averages of the RC-LH complex (top) and corresponding raw particles (bottom). **D,** The angular distribution of RC-LH complex particles in the final reconstruction. The direction and length of each cylinder represent the direction and the amount of particles. **E,** Resolution estimation. The gold-standard FSC curve between two independent maps and the FSC curve between independent phase-randomized maps are shown in red and green, respectively. The FSC curve between the map and the final refined model is shown in blue. **F,** Local resolution of the cryo-EM map estimated by ResMap [1]. All structural figures here were generated with UCSF Chimera [2].

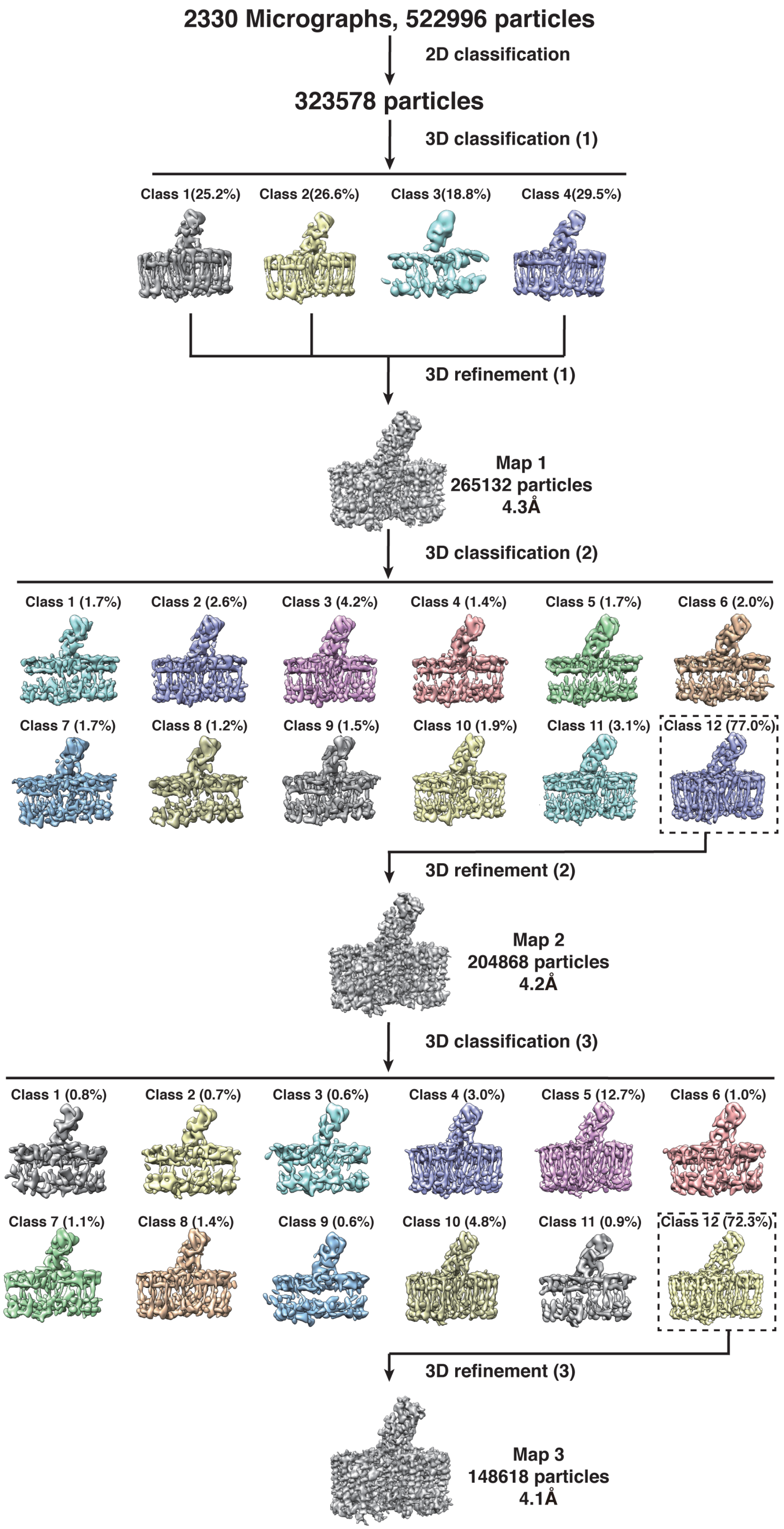

2330 Micrographs, 522996 particles
2D classification
323578 particles
3D classification (1)
Class 1(25.2%)
Class 2(26.6%)
Class 3(18.8%)
Class 4(29.5%)
3D refinement (1)
Map 1
265132 particles
4.3Å
3D classification (2)
Class 1 (1.7%)
Class 2 (2.6%)
Class 3 (4.2%)
Class 4 (1.4%)
Class 5 (1.7%)
Class 6 (2.0%)
Class 7 (1.7%)
Class 8 (1.2%)
Class 9 (1.5%)
Class 10 (1.9%)
Class 11 (3.1%)
Class 12 (77.0%)
3D refinement (2)
Map 2
204868 particles
4.2Å
3D classification (3)
Class 1 (0.8%)
Class 2 (0.7%)
Class 3 (0.6%)
Class 4 (3.0%)
Class 5 (12.7%)
Class 6 (1.0%)
Class 7 (1.1%)
Class 8 (1.4%)
Class 9 (0.6%)
Class 10 (4.8%)
Class 11 (0.9%)
Class 12 (72.3%)
3D refinement (3)
Map 3
148618 particles
4.1Å

Supplementary Figure 3 | The procedure for cryo-EM data processing. A total of 522,996 particles imaged on FEI Falcon IIIEC camera were subjected to reference-free 2D classification. After removal of bad classes, 323,578 particles were selected for 3D classification. The first round of 3D classification divided the particles into 4 classes, and 3 classes of them (265,132 particles) with better overall feature were combined for refinement, which yield a map at 4.3 Å resolution (map 1). The second round 3D classification was performed based on refinement of map 1 without any alignments, which divided the particles into 12 classes. Only 1 class of them (204,868 particles) was selected for refinement and a map at 4.2 Å resolution (map 2) was obtained. To further improve the reconstruction, the third round 3D classification and refinement were performed with the same strategy as the second round, and the resolution of the final map was 4.1 Å resolution (map 3). Additional cycles of 3D classifications and refinements did not further improve the overall resolution of the map. All structural figures here were generated with UCSF Chimera [2].

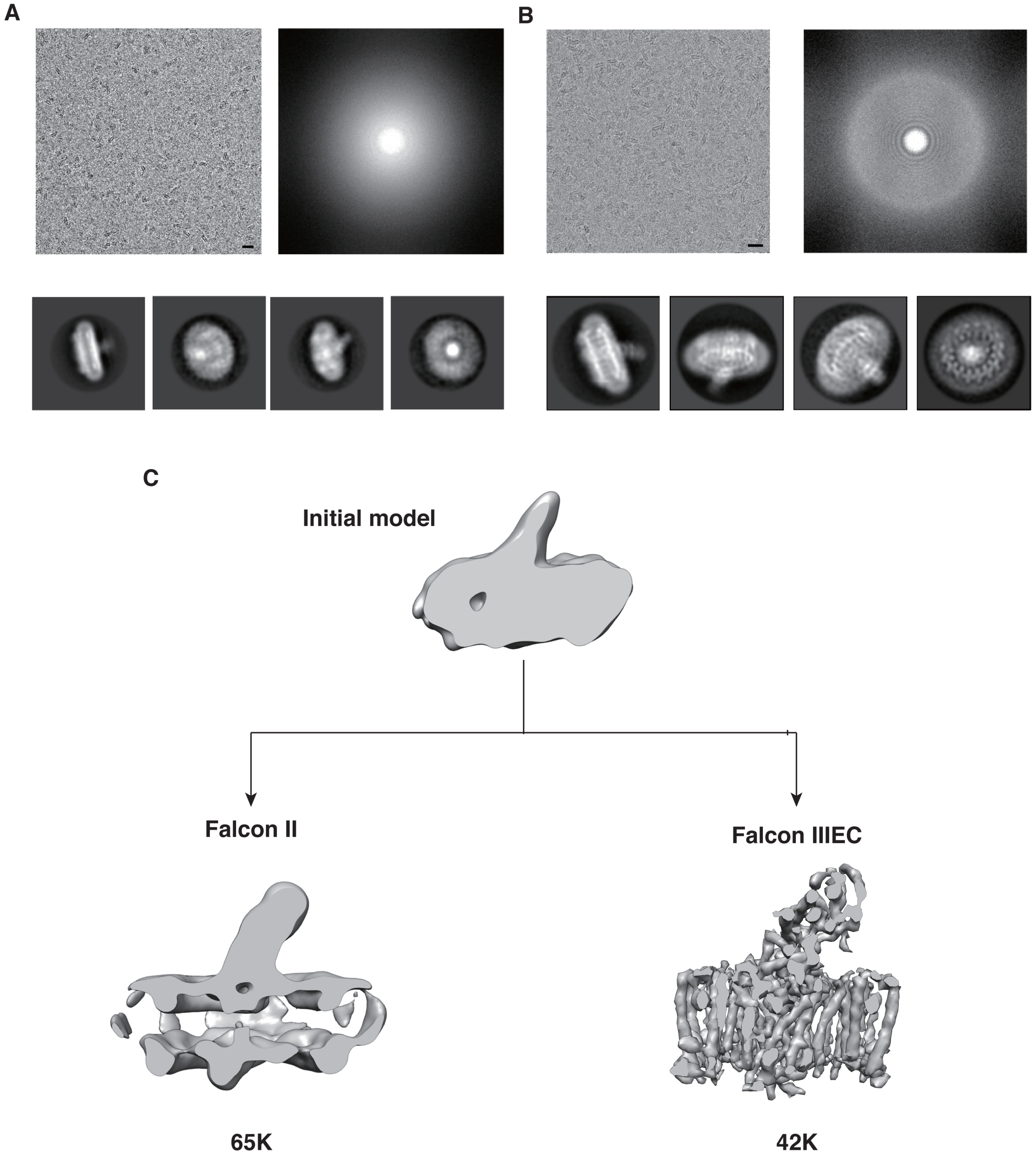

Supplementary Figure 4 | Comparative analysis of the images acquired on FEI Falcon II and Falcon IIIEC cameras. **A,** Analysis of RC-LH complex imaged on Falcon II camera. A representative raw electron micrograph, its power spectrum and representative reference-free 2D class averages are

show at left-top, right-top and bottom panels, respectively. Scale bar, 20 nm. **B,** Analysis of RC-LH complex imaged on Falcon IIIEC camera. Scale bar, 20 nm. **C,** Comparison of densities reconstructed from images acquired on two cameras. With the same initial model and fewer number of particles, the map reconstructed from the data acquired from Falcon IIIEC camera represents higher resolution than that from Falcon II camera. All structural figures here were generated with UCSF Chimera [2].

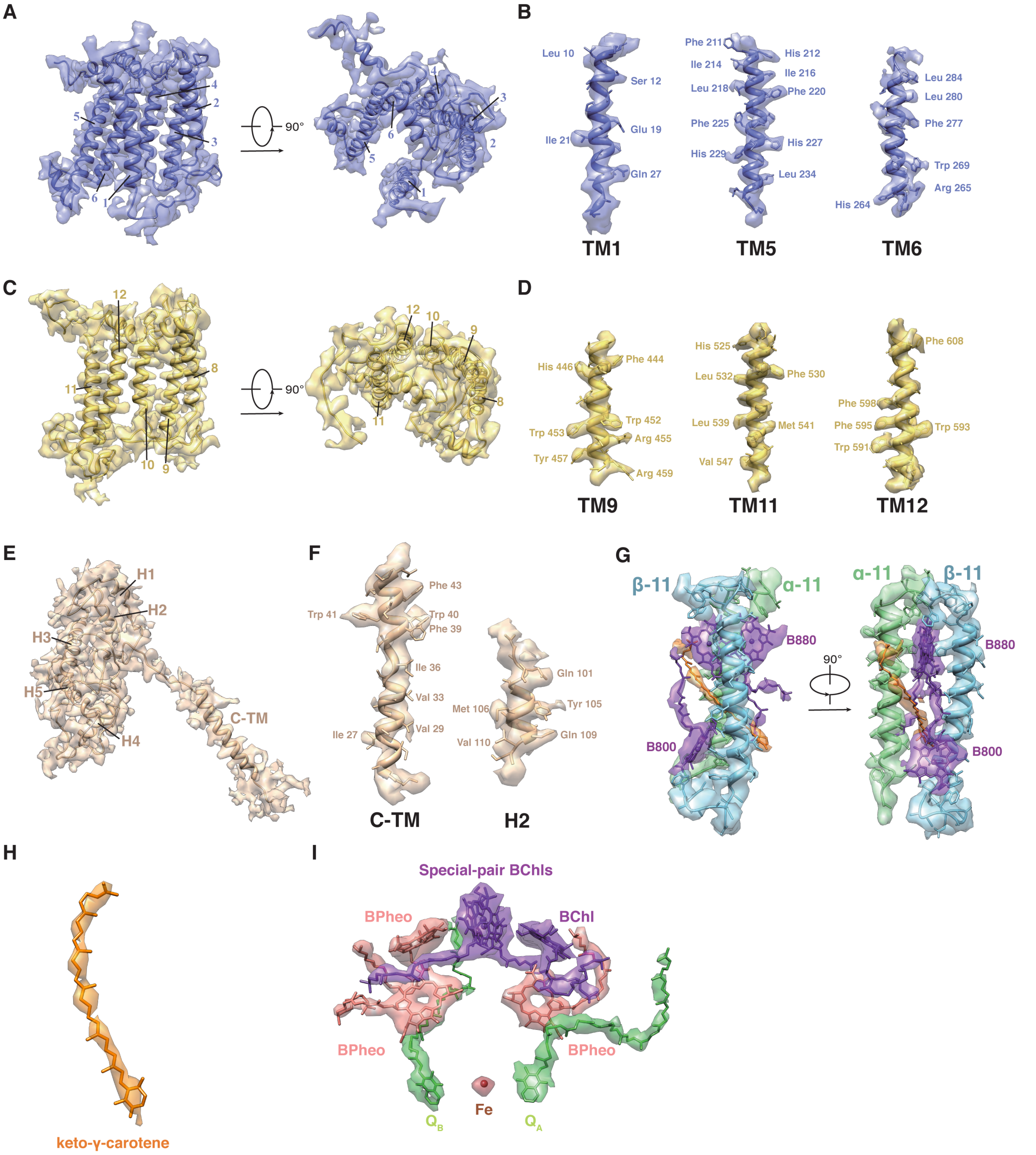

Supplementary Figure 5 | Close-up views of cryo-EM density maps of *rc*RC-LH complex. A, The density map docked with the model (backbone chain trace) of L subunit in the side (left) and top (right) view. **B,** EM density maps and atomic models are shown for the TM1, TM5 and TM6 of L subunit. **C,**

The density map docked with the model (backbone chain trace) of M subunit in the side (left) and top (right) view. **D,** EM density maps and atomic models are shown for the TM9, TM11 and TM12 of M subunit. **E,** The density map docked with the model (backbone chain trace) of Cyt *c* subunit. **F,** EM density maps and atomic models are shown for the helix C-TM and H2 of Cyt *c*. **G,** EM density map and atomic model are shown for the the 11th αβ-heterodimer with three BChls and one γ-carotenoid in two side views with an interval of 90°. **H,** A representative density map of keto-γ-carotene fitted with model. **I,** EM density map of the cofactors in L and M subunits docked by the model. The color codes are same as **Figure 1**. All structural figures here were generated with UCSF Chimera [2].

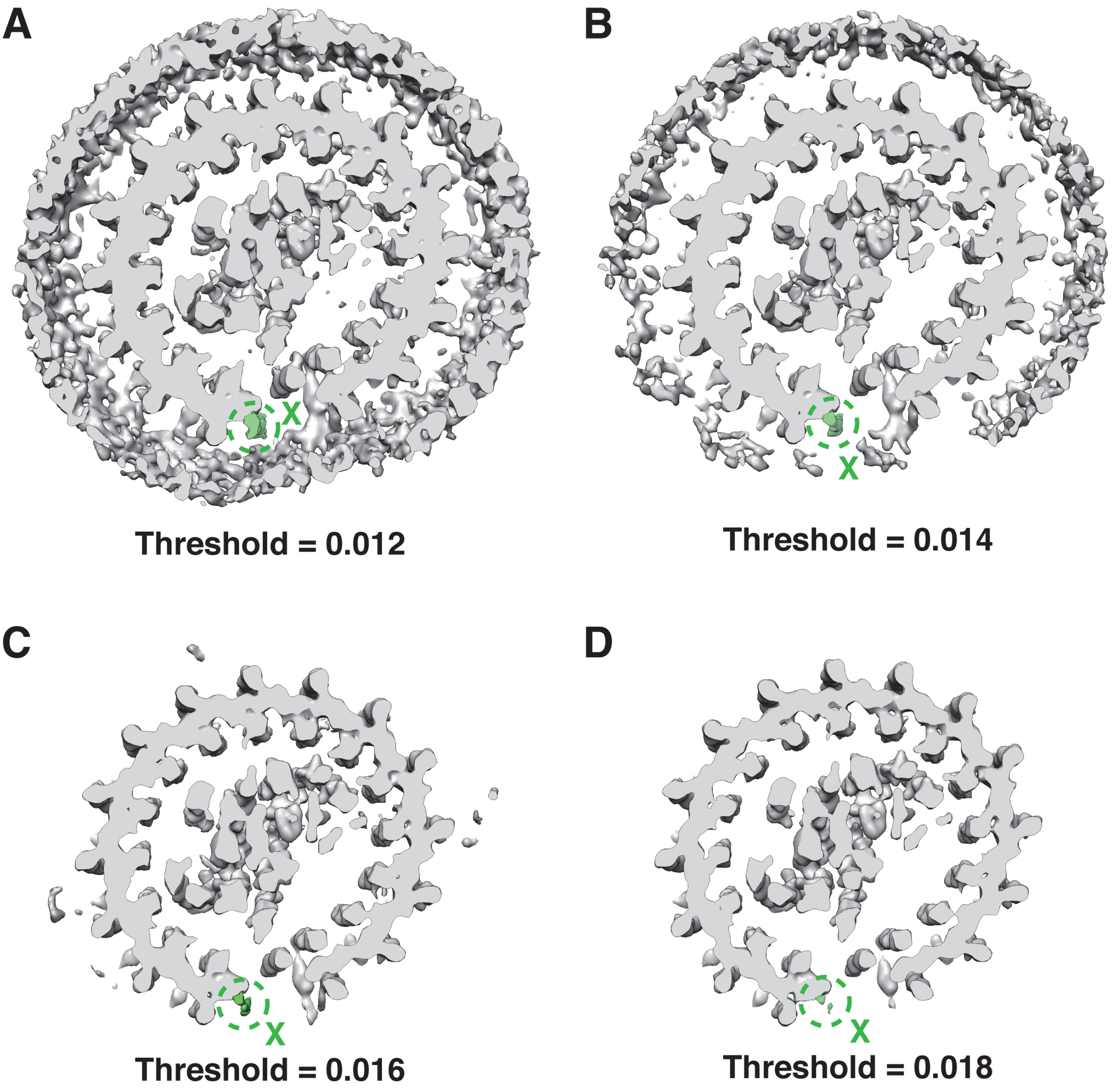

Supplementary Figure 6 | Visualization of subunit X and its surrounding densities at different thresholds. A low-pass (6 Å) filtered map was visualized at different thresholds of 0.012 (**A**), 0.014 (**B**), 0.016 (**C**), and 0.018 (**D**), respectively. The subunit X was shown as green and highlighted with a dashed circle. All structural figures here were generated with UCSF Chimera [2].

A **L and M**

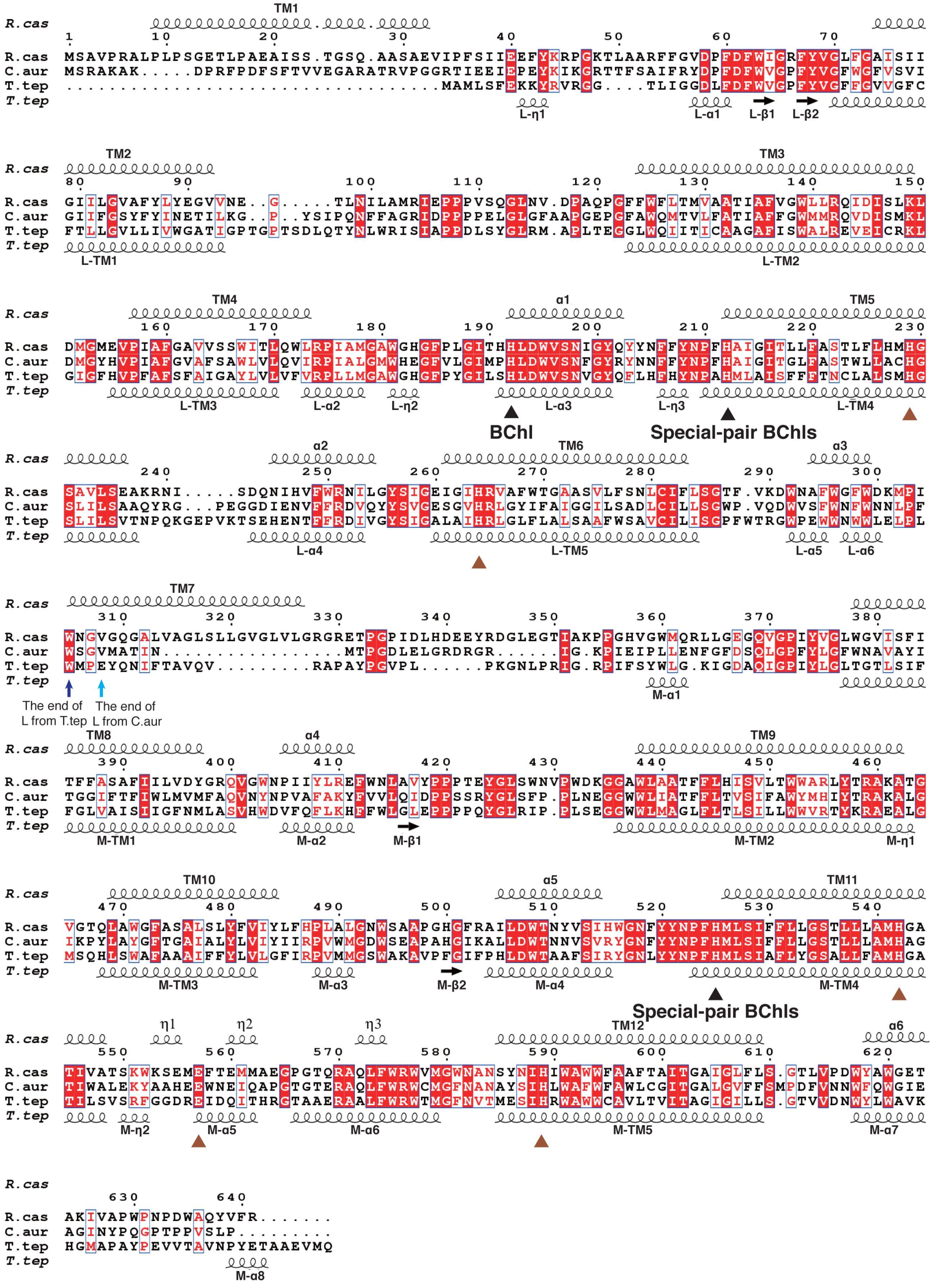
TM1
R.cas MSAVPRALPLPSGETLPAEAISS.TGSQ.AASAEVIPFSIIEEFYKRPGKTLAARFFGVDPFDFWIGRFYVGLFGAISII
C.aur MSRAKAK.....DPRFPDFSFTVVEGARATRVPGGRTIEEIEPEYKIKGRTTFSAIFRYDPFDFWVGPFYVGFWGFVSVI
T.tep ...............................MAMLSFEKKYRVRGG....TLIGGDLFDFWVGPFYVGFFGVVGFC
L-η1 L-α1 L-β1 L-β2
TM2 TM3
R.cas GIILGVAFYLYEGVVNE..G.....TLNILAMRIEPPPVSQGLNV.DPAQPGFFWFLTMVAATIAFVGWLLRQIDISLKL
C.aur GIIFGSYFYINETILKG..P..YSIPQNFFAGRIDPPPPELGLGFAAPGEPGFAWQMTVLFATIAFFGWMMRQVDISMKL
T.tep FTLLGVLLIVWGATIGPTGPTSDLQTYNLWRISIAPPDLSYGLRM.APLTEGGLWQIITICAAGAFISWALREVEICRKL
L-TM1 L-TM2
TM4 α1 TM5
R.cas DMGMEVPIAFGAVVSSWITLQWLRPIAMGAWGHGFPLGITHHLDWVSNIGYQYYNFFYNPFHAIGITLLFASTLFLHMHG
C.aur DMGYHVPIAFGVAFSAWLVLQVIRPIALGMWHEGFVLGIMPHLDWVSNFGYRYNNFFYNPFHAIGITGLFASTWLLACHG
T.tep GIGFHVPFAFSFAIGAYLVLVFVRPLLMGAWGHGFPYGILSHLDWVSNVGYQFLHFHYNPAHMLAISFFFTNCLALSMHG
L-TM3 L-α2 L-η2 L-α3 L-η3 L-TM4
BChl
Special-pair BChls
α2 TM6 α3
R.cas SAVLSEAKRNI.....SDQNIHVFWRNILGYSIGEIGIHRVAFWTGAASVLFSNLCIFLSGTF.VKDWNAFWGFWDKMPI
C.aur SLILSAAQYRG...PEGGDIENVFFRDVQYYSVGESGVHRLGYIFAIGGILSADLCILLSGWP.VQDWVSFWNFWNNLPF
T.tep SLILSVTNPQKGEPVKTSEHENTFFRDIVGYSIGALAIHRLGLFLALSAAFWSAVCILISGPFWTRGWPEWWNWWLELPL
L-α4 L-TM5 L-α5 L-α6
TM7
R.cas WNGVGQGALVAGLSLLGVGLVLGRGRETPGPIDLHDEEYRDGLEGTIAKPPGHVGWMQRLLGEGQVGPIYVGLWGVISFI
C.aur WSGVMATIN.................MTPGDLELGRDRGR......IG.KPIEIPLLENFGFDSQLGPFYLGFWNAVAYI
T.tep WMPEYQNIFTAVQV.........RAPAYPGVPL......PKGNLPRIG.RPIFSYWLG.KIGDAQIGPIYLGLTGTLSIF
M-α1
The end of L from T.tep
The end of L from C.aur
TM8 α4 TM9
R.cas TFFASAFIILVDYGRQVGWNPIIYLREFWNLAVYPPPTEYGLSWNVPWDKGGAWLAATFFLHISVLTWWARLYTRAKATG
C.aur TGGIFTFIWLMVMFAQVNYNPVAFAKYFVVLQIDPPSSRYGLSFP.PLNEGGWWLIATFFLTVSIFAWYMHIYTRAKALG
T.tep FGLVAISIIGFNMLASVHWDVFQFLKHFFWLGLEPPPPQYGLRIP.PLSEGGWWLMAGLFLTLSILLWWVRTYKRAEALG
M-TM1 M-α2 M-β1 M-TM2 M-η1
TM10 α5 TM11
R.cas VGTQLAWGFASALSLYFVIYLFHPLALGNWSAAPGHGFRAILDWTNYVSIHWGNFYYNPFHMLSIFFLLGSTLLLAMHGA
C.aur IKPYLAYGFTGAIALYLVIYIIRPVWMGDWSEAPAHGIKALLDWTNNVSVRYGNFYYNPFHMLSIFFLLGSTLLLAMHAG
T.tep MSQHLSWAFAAAIFFYLVLGFIRPVMMGSWAKAVPFGIFPHLDWTAAFSIRYGNLYYNPFHMLSIAFLYGSALLFAMHGA
M-TM3 M-α3 M-β2 M-α4 M-TM4
Special-pair BChls
η1 η2 η3 TM12 α6
R.cas TIVATSKWKSEMEFTEMMAEGPGTQRAQLFWRWVMGWNANSYNIHIWAWWFAAFTAITGAIGLFLS.GTLVPDWYAWGET
C.aur TIWALEKYAAHEEWNEIQAPGTGTERAQLFWRWCMGFNANAYSIHLWAFWFAWLCGITGALGVFFSMPDFVNNWFQWGIE
T.tep TILSVSRFGGDREIDQITHRGTAAERAALFWRWTMGFNVTMESIHRWAWWCAVLTVITAGIGILLS.GTVVDNWYLWAVK
M-η2 M-α5 M-α6 M-TM5 M-α7
R.cas AKIVAPWPNPDWAQYVFR.......
C.aur AGINYPQGPTPPVSLP.........
T.tep HGMAPAYPEVVTAVNPYETAAEVMQ
M-α8

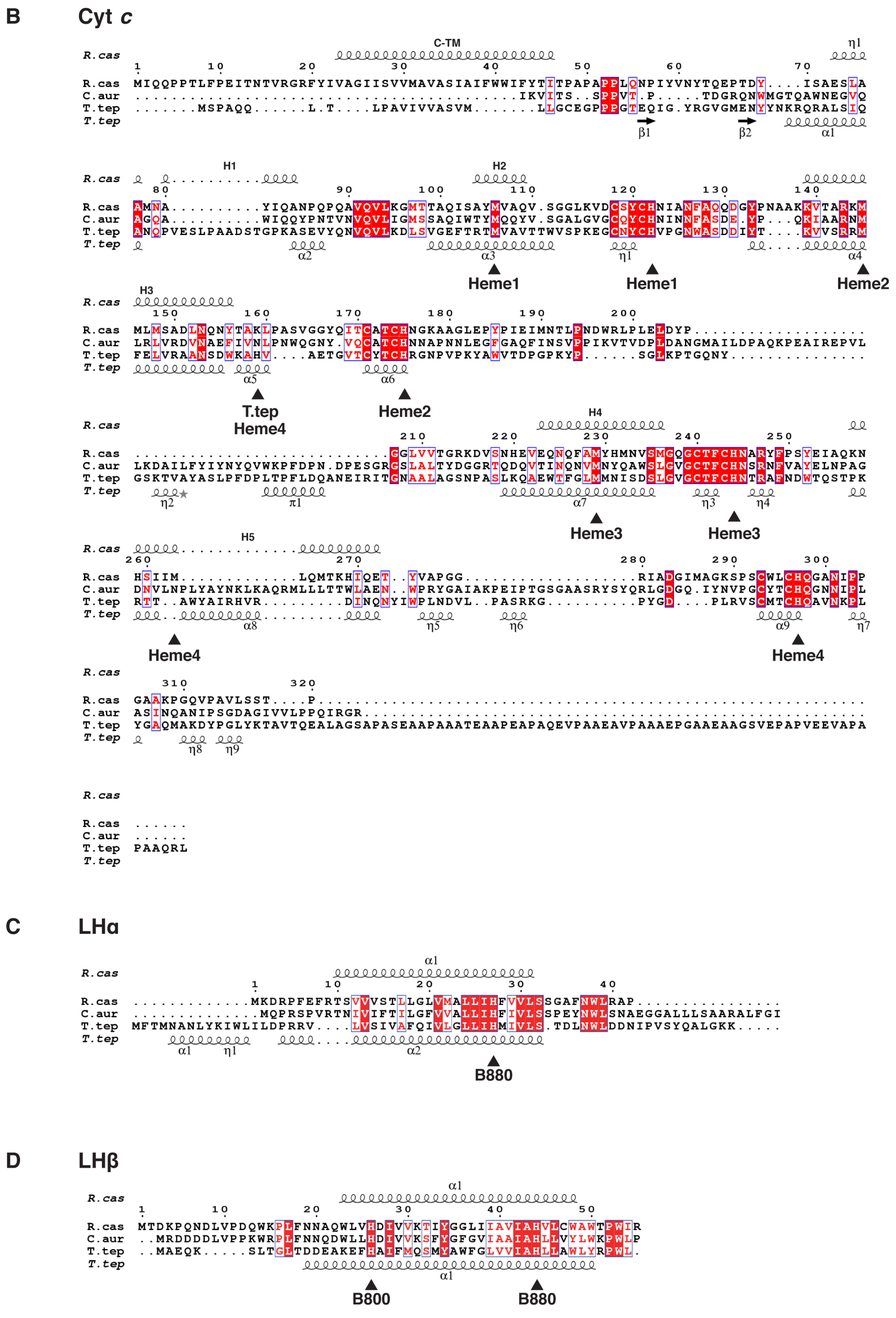

B
Cyt c
R.cas
C-TM
η1
1 10 20 30 40 50 60 70
R.cas MIQQPPTLFPEITNTVRGRFYIVAGIISVVMAVASIAIFWWIFYTITPAPAPPLQNPIYVNYTQEPTDY....ISAESLA
C.aur ....IKVITS..SPPVT.P.....TDGRQNWMGTQAWNEGVQ
T.tep .......MSPAQQ......L.T....LPAVIVVASVM.......LLGCEGPPPGTEQIG.YRGVGMENYYNKRQRALSIQ
T.tep
β1 β2 α1
H1 H2
R.cas
80 90 100 110 120 130 140
R.cas AMNA..........YIQANPQPQAVQVLKGMTTAQISAYMVAQV.SGGLKVDCSYCHNIANFAQQDGYPNAAKKVTARKM
C.aur AGQA..........WIQQYPNTVNVQVLIGMSSAQIWTYMQQYV.SGALGVGCQYCHNINNFASDE.YP...QKIAARNM
T.tep ANQPVESLPAADSTGPKASEVYQNVQVLKDLSVGEFTRTMVAVTTWVSPKEGCNYCHVPGNWASDDIYT....KVVSRRM
T.tep
α2 α3 η1 α4
Heme1
Heme1
Heme2
H3
R.cas
150 160 170 180 190 200
R.cas MLMSADLNQNYTAKLPASVGGYQITCATCHNGKAAGLEPYPIEIMNTLPNDWRLPLELDYP..................
C.aur LRLVRDVNAEFIVNLPNWQGNY.VQCATCHNNAPNNLEGFGAQFINSVPIKVTVDPLDANGMAILDPAQKPEAIREPVL
T.tep FELVRAANSDWKAHV....AETGVTCYTCHRGNPVPKYAWVTDPGPKYP......SGLKPTGQNY..............
T.tep
α5 α6
T.tep Heme4
Heme2
H4
R.cas
210 220 230 240 250
R.cas ...............................GGLVVTGRKDVSNHEVEQNQFAMYHMNVSMGQGCTFCHNARYFPSYEIAQKN
C.aur LKDAILFYIYNYQVWKPFDPN.DPESGRGSLALTYDGGRTQDQVTINQNVMNYQAWSLGVGCTFCHNSRNFVAYELNPAG
T.tep GSKTVAYASLPFDPLTPFLDQANEIRITGNAALAGSNPASLKQAEWTFGLMMNISDSLGVGCTFCHNTRAFNDWTQSTPK
T.tep
η2 π1 α7 η3 η4
Heme3
Heme3
H5
R.cas
260 270 280 290 300
R.cas HSIIM.............LQMTKHIQET..YVAPGG...................RIADGIMAGKSPSCWLCHQGANIPP
C.aur DNVLNPLYAYNKLKAQRMLLLTTWLAEN..WPRYGAIAKPEIPTGSGAASRYSYQRLGDGQ.IYNVPGCYTCHQGNNIPL
T.tep RTT..AWYAIRHVR.........DINQNYIWPLNDVL..PASRKG.........PYGD....PLRVSCMTCHQAVNKPL
T.tep
α8 η5 η6 α9 η7
Heme4
Heme4
R.cas
310 320
R.cas GAAKPGQVPAVLSST....P...
C.aur ASINQANIPSGDAGIVVLPPQIRGR..
T.tep YGAQMAKDYPGLYKTAVTQEALAGSAPASEAAPAAATEAAPEAPAQEVPAAEAVPAAAEPGAAEAAGSVEPAPVEEVAPA
T.tep
η8 η9
R.cas
R.cas ......
C.aur ......
T.tep PAAQRL
T.tep
C
LHα
α1
R.cas
1 10 20 30 40
R.cas .............MKDRPFEFRTSVVVSTLLGLVMALLIHFVVLSSGAFNWLRAP................
C.aur ..............MQPRSPVRTNIVIFTILGFVVALLIHFIVLSSPEYNWLSNAEGGALLLSAARALFGI
T.tep MFTMNANLYKIWLILDPRRV....LVSIVAFQIVLGLLIHMIVLS.TDLNWLDDNIPVSYQALGKK.....
T.tep
α1 η1 α2
B880
D
LHβ
α1
R.cas
1 10 20 30 40 50
R.cas MTDKPQNDLVPDQWKPLFNNAQWLVHDIVVKTIYGGLIIAVIAHVLCWAWTPWIR
C.aur ..MRDDDDLVPPKWRPLFNNQDWLLHDIVVKSFYGFGVIAAIAHLLVYLWKPWLP
T.tep ..MAEQK.....SLTGLTDDEAKEFHAIFMQSMYAWFGLVVIAHLLAWLYRPWL.
T.tep
α1
B800
B880

Supplementary Figure 7 | The structure based sequence alignment. Sequence alignment of LM (the sequences of *C. aurantiacus* and T. *tepidum* are artificially spliced) subunits (**A**), Cyt *c* (**B**), LHα (**C**) and LHβ (**D**). Fully conserved residues are shaded by red, and highly conserved residues are shown as red characters. The secondary structure depiction of *rc*RC-LH *complex* and *tt*RC-LH1 *complex* are shown at the top and bottom, respectively. The residues coordinate iron molecules in the LM subunits are labelled by brown triangles. The sequence alignments were calculated by Clustal Omega [3] and presented here by ESPript [4].

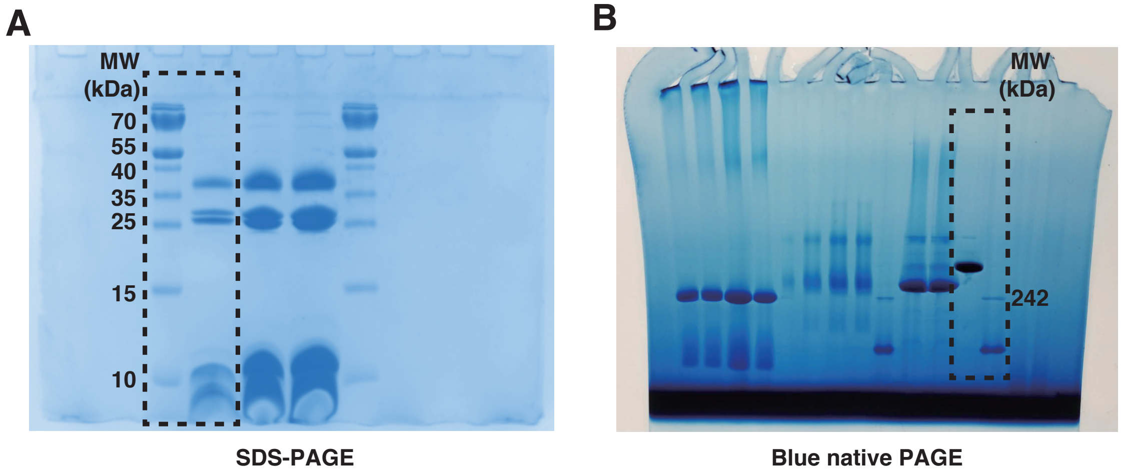

Supplementary Figure 8 |. Uncropped and unprocessed images of the gels. A, SDS-PAGE gel of LH-RC in different concentration. **B,** Blue native PAGE gel of *rc*RC-LH. The dashed boxes indicate the lanes that were displayed in Supplementary Figure 1A.

Subunit	Accession	Description	Score	Coverage
L subunit	BAC76414.1	precursor for L and M subunits of photosynthetic reaction center [*Roseiflexus castenholzii*]	166	31%
M subunit	BAC76414.1	precursor for L and M subunits of photosynthetic reaction center [*Roseiflexus castenholzii*]	109	19%
Cyt *c*	BAC76415.1	cytochrome subunit of photosynthetic reaction center [*Roseiflexus castenholzii*]	395	15%
α subunit	BAC76415.1	cytochrome subunit of photosynthetic reaction center [*Roseiflexus castenholzii*]	8.21	6.56%
	BAC76413.1	alpha subunit of light-harvesting 1 [*Roseiflexus castenholzii*]	7.05	21.43%
	WP_041331144.1	hypothetical protein (X) [*Roseiflexus castenholzii*]	3.72	38.10%
β subunit	BAC76412.1	beta subunit of light-harvesting 1 [Roseiflexus castenholzii]	7.21	30.91%
X subunit	WP_041331144.1	hypothetical protein (X) [Roseiflexus castenholzii]	125.67	38.10%
	BAC76415.1	cytochrome subunit of photosynthetic reaction center [Roseiflexus castenholzii]	16.20	14.06%
	WP_012120605.1	NADH-quinone oxidoreductase subunit NuoK [Roseiflexus castenholzii]	10.84	13.89%

Supplementary Table 1 | Peptide mass fingerprinting (PMF) analysis of each subunit of the RC-LH complex from SDS-PAGE.

Accession	Description	Score	Coverage
BAC76415.1	cytochrome subunit of photosynthetic reaction center [Roseiflexus castenholzii]	1571.83	45.00%
BAC76414.1	precursor for L and M subunits of photosynthetic reaction center [Roseiflexus castenholzii]	570.29	18.72%
WP_012119986.1	4Fe-4S dicluster domain-containing protein [Roseiflexus castenholzii]	72.41	15.54%
WP_041331144.1	hypothetical protein (X) [Roseiflexus castenholzii]	53.80	38.10%
BAC76413.1	alpha subunit of light-harvesting 1 [Roseiflexus castenholzii]	36.66	21.43%
WP_012121558.1	FAD-binding protein [Roseiflexus castenholzii]	33.11	15.70%
BAC76412.1	beta subunit of light-harvesting 1 [Roseiflexus castenholzii]	20.52	27.27%

Supplementary Table 2 | Peptide mass fingerprinting (PMF) analysis of the RC-LH complex from blue native PAGE.

	RC-LH (EMDB-6828) (PDB 5YQ7)
**Data collection and processing**	
Magnification	75,000
Voltage (kV)	300
Electron exposure (e–/Å^2)	40
Defocus range (μm)	1.0 to 3.5
Pixel size (Å)	1.12
Symmetry imposed	C1
Initial particle images (no.)	522,996
Final particle images (no.)	148,618
Map resolution (Å)	4.1
FSC threshold	0.143
**Refinement**	
Initial model used (PDB code)	3wmm
Model resolution (Å)	4.4
FSC threshold	0.5
Map sharpening *B* factor (Å^2)	-100
Model composition	
Non-hydrogen atoms	20,002
Protein residues	2155
Ligands	72
R.m.s. deviations	
Bond lengths (Å)	0.01
Bond angles (°)	1.55
Validation	
MolProbity score	2.35
Clashscore	15.34
Poor rotamers (%)	0.14
Ramachandran plot	
Favored (%)	85.27
Allowed (%)	14.54
Disallowed (%)	0.19

Supplementary Table 3 | Cryo-EM data collection, refinement and validation statistics.

	1	2	3	4	5	6	7	8	9	10	11	12	13	14	15
B800	-	3.3	3.1	4.2	4.0	3.1	3.0	3.0	3.0	3.0	3.4	3.3	2.9	3.1	-
B880	-	3.7	4.0	3.7	4.8	3.6	3.5	3.5	3.6	4.2	3.8	4.2	3.8	4.6	3.8

Supplementary Table 4 | Edge to edge distance (Å) of carotenoid to B800/B880

	1	2	3	4	5	6	7	8	9	10	11	12	13	14	15
edge to edge*	3.8	3.8	3.3	3.5	3.5	3.7	3.6	3.7	3.5	3.3	3.4	3.4	3.4	4.0	4.0
edge to edge†	3.8	4.9	4.3	3.7	4.4	5.1	4.5	4.7	4.4	4.4	4.3	4.5	4.2	4.8	20.5
Mg to Mg*	9.6	8.9	9.1	9.8	9.3	9.3	9.7	9.4	9.6	9.4	9.8	9.5	9.5	9.9	10.1
Mg to Mg†	9.4	9.7	9.5	8.8	9.5	9.5	9.3	9.4	9.3	9.4	9.1	9.1	9.3	8.8	27.7

Supplementary Table 5 | The distances (Å) between adjacent B880.

*distance between the B880 bound by the same transmembrane pairs of LH.

†distance between the B880 bound by adjacent transmembrane pairs of LH.

	1	2	3	4	5	6	7	8	9	10	11	12	13	14	15	16
*rc*RC-LH	13.1	11.7	11.7	10.4	11.1	12.6	13.1	14.7	12.6	14.8	12.5	11.8	12.2	10.7	7.8	-
*tt*RC-LH1	8.4	9.0	8.0	9.3	8.6	8.2	8.7	8.6	8.5	9.5	10.4	8.6	10.3	11.7	8.8	6.8

Supplementary Table 6 | The angels (°) between helices in each transmembrane pair.

	1	2	3	4	5	6	7	8	9	10	11	12	13	14	15*	16†
Alpha	14.5	15.2	15.1	14.4	14.2	14.9	15.2	14.1	15.3	14.2	14.9	15.3	14.4	14.5	18.4	9.8
Beta	19.9	19.6	20.0	19.9	20.0	20.6	19.7	19.8	19.7	19.6	19.8	19.9	19.8	20.0	10.8	27.8

Supplementary Table 7 | The distance (Å) between helixes of inter-subunit in *rc*RC-LH complex.

The distance is measured between LHαβn and LHαβ (n+1).
* the distances from LHα15 to C-TM and LHβ15 to subunit X.
† the distances from LHα1 to C-TM and LHβ1 to subunit X.

References

1. Kucukelbir, A., Sigworth, F.J. & Tagare, H.D. Quantifying the local resolution of cryo-EM density maps. *Nat Methods* **11**, 63-5 (2014).
2. Pettersen, E.F. et al. UCSF Chimera--a visualization system for exploratory research and analysis. *J Comput Chem* **25**, 1605-12 (2004).
3. Sievers, F. et al. Fast, scalable generation of high-quality protein multiple sequence alignments using Clustal Omega. in *Molecular systems biology* Vol. 7 539 (2011).
4. Robert, X. & Gouet, P. Deciphering key features in protein structures with the new ENDscript server. *Nucleic Acids Research* **42**, W320-W324 (2014).
