# Supplementary material for: Cryo-EM structure of the RC-LH core complex from an early branching photosynthetic prokaryote
Source: Nat Commun. 2018 Apr 19;9:1568. doi: 10.1038/s41467-018-03881-x (PMC5908803; doi:10.1038/s41467-018-03881-x)
Supplement: Supplementary file 3 — Description of Additional Supplementary Files [file 41467_2018_3881_MOESM3_ESM.pdf]

Description of Additional Supplementary Files

File Name: Supplementary Movie 1
Description: The overall map with model fitted in a slicing view.

File Name: Supplementary Movie 2
Description: The overall low-pass (6 angstrom) filtered map with model fitted in a slicing view.
